# Measured Composite Collision Models: Quantum Trajectory Purities and Channel Divisibility

**DOI:** 10.3390/e24050715

**Published:** 2022-05-17

**Authors:** Konstantin Beyer, Kimmo Luoma, Tim Lenz, Walter T. Strunz

**Affiliations:** 1 Institut für Theoretische Physik, Technische Universität Dresden, D-01062 Dresden, Germany; tim.lenz@tu-dresden.de (T.L.); walter.strunz@tu-dresden.de (W.T.S.); 2 Laboratory of Quantum Optics and Turku Center for Quantum Physics, Department of Physics and Astronomy, University of Turku, FI-20014 Turun Yliopisto, Finland; ktluom@utu.fi

**Keywords:** collision models, quantum measurements, non-Markovian quantum dynamics

## Abstract

We investigate a composite quantum collision model with measurements on the memory part, which effectively probe the system. The framework allows us to adjust the measurement strength, thereby tuning the dynamical map of the system. For a two-qubit setup with a symmetric and informationally complete measurement on the memory, we study the divisibility of the resulting dynamics in dependence of the measurement strength. The measurements give rise to quantum trajectories of the system and we show that the average asymptotic purity depends on the specific form of the measurement. With the help of numerical simulations, we demonstrate that the different performance of the measurements is generic and holds for almost all interaction gates between the system and the memory in the composite collision model. The discrete model is then extended to a time-continuous limit.

## 1. Introduction

Collision models are a versatile tool for investigating open quantum system dynamics (for examle, see the recent review [1]). They have been used to explore quantum memory effects [2,3,4,5,6,7], quantum thermodynamics [8,9,10,11,12,13,14,15,16], and system–environment correlations [10,17,18]. In this article, we focus on a so-called composite collision model. In such a model, the system of interest interacts sequentially with a memory. The latter, in turn, interacts with a sequence of environmental ancillas that are uncorrelated before and traced out after their collision. By construction, a composite collision model explicitly allows for the inclusion of possible memory effects of the environment into the dynamics. The dynamics of the system of interest is then defined by tracing out the memory. It has been shown that models of this type can, in general, lead to a non-Markovian behaviour in the system and that they can efficiently mimic memory effects in the environment known from microscopic open quantum system models [1,4,19].

Collision models often focus on the description of the reduced dynamics emerging in the system. In this work, we will employ a quantum trajectory approach to analyse which knowledge one can gain about the system when measurements are performed on the memory part of the composite model. In particular, we ask how pure the conditional states in the system can become on average. In a standard Markovian collision model without memory, one can always obtain pure state trajectories in physically valid schemes [20]. In a time-continuous limit, this can lead, e.g., to a Markovian unravelling of a Gorini–Kossakowski–Sudarshan–Lindblad (GKSL) equation into a stochastic Schrödinger equation for pure states [21,22].

If memory effects are included, the situation is less clear. There are stochastic Schrödinger equation approaches that are able to describe non-Markovian dynamics through an ensemble of pure states such as, e.g., the non-Markovian quantum state diffusion (NMQSD) [23]. In most cases a continuous measurement interpretation for a physical quantum trajectory is missing [24,25,26,27,28]. The success of these methods is based on the fact that they can efficiently solve the reduced dynamics of the system. Recently, however, a non-Markovian mixed state trajectory theory has been established [29].

Composite collision models provide an ideal framework to discuss these issues. In this contribution, we consider a composite collision model consisting of two qubits and study the role of different measurement schemes and different measurement strengths in the memory part. By construction, this setup generates physical quantum trajectories. Clearly, although the considered measurement schemes realise the same dynamical map, the resulting trajectories of the system can be quite different. In particular, the purity of these trajectories strongly depends on the performed measurement on the memory. Crucially, the trajectories of the joint state of system and memory can always be chosen to be pure, since the behaviour of the joint state is Markovian. Thus, the non-pure states in the system trajectories arise solely from the entanglement between system and memory. Stronger measurements on the memory part reduce the quantum correlations with the system part, and therefore allow for purer trajectories in the latter. On the other hand, on average, stronger measurements also result in more disturbance and lead to a faster degradation of the reduced state of the memory. As we will see, this in turn counteracts a non-Markovian behaviour of the reduced dynamics in the system alone. Thus, generally there is a trade-off between the non-Markovianity of the reduced dynamics and the purity of the quantum trajectories. This can be understood by the fact that the more information a measurement provides, the more it has to disturb the state on average.

In order to show that the different measurement strengths and schemes perform differently in a generic situation, we sample collision models by randomly choosing the interaction gate between the system and the memory. This ensures that the observed relationship between the measurement strength and the non-Markovian behaviour, as well as the different purities of the quantum trajectories for different measurement schemes, do not stem from an accidental fine-tuning between the measurement and the interaction, but hold in general.

The article is structured as follows. In Section 2, we present our measured composite collision model and discuss relevant concepts needed in the remainder: channel divisibility, quantum trajectories arising from underlying instruments, and their ensemble purities. In the following Section 3, we describe a specific case and analyse its generic behaviour using randomly sampled gates in a discrete collision model. We then show in Section 4 that a time-continuous limit can be established and we compare the results to the discrete case. We conclude in Section 5 with a brief outlook.

## 2. The Model

**Figure 1:**
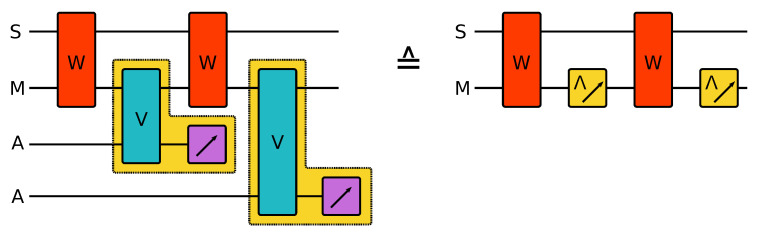
The composite collision model. The system S interacts repeatedly with the same memory system M. The memory M subsequently interacts with a fresh ancilla in each step. If only the reduced dynamics of S and M is of interest, the ancillas can be traced out after their collision. However, here we consider a model where the ancillas are measured after the interaction, which allows us to obtain some information about the current state of S and M. This indirect measurement can be seen as a dilation of a direct measurement on M, and therefore, the model can be given in an equivalent but more compact form without an explicit reference to the ancillas. We will use the latter description in this article. Discarding the outcome, the measurements constitute a channel Λ.

We consider a measured composite collision model as presented schematically in Figure 1. In each step, the system S and the memory M interact by a unitary gate *W*. Subsequently, a measurement is performed on the memory part. Such a measurement can always be described as a coupling to an ancilla system and a subsequent measurement of the latter. However, since we are not concerned with such an explicit dilation here, we keep the notation simple and describe the measurements directly on M (see Figure 1 for details). In this article, we focus on finite quantum systems and measurements with a finite number of outcomes. In general, an observable is given by a positive operator valued measure (POVM), i.e., a collection of positive operators $E_{i}>0$, which sum to unity $\sum_{i}E_{i}=1$, where *i* labels the measurement outcome [30]. Since the post-measurement state is fed back into the circuit, the measurement process itself must be considered in detail. The state transformation due to the measurement is given by a quantum operation $\rho_{M}\mapsto \rho_{M}^{i}=I_{i}\left(\rho_{M}\right)=\sum_{k}M_{ik}\;\rho_{M}\;M_{ik}^{†}$ such that $p_{i}=\mathrm{tr}\left[\rho_{M}E_{i}\right]=\sum_{k}\mathrm{tr}\left[M_{ik}\;\rho_{M}\;M_{ik}^{†}\right]$ for any state $\rho_{M}$ of the memory. We call $I_{i}$ the instrument implementing the observable $E_{i}$. It can be shown that there is no instrument that leaves all states of the system unchanged unless the associated observable is trivial [31]. The measurement is called efficient if there is only one measurement operator $M_{ik}$ for each outcome *i* [32]. Inefficient measurements will lead to additional noise and mixed trajectories, even for the joint state of the system and the memory. Therefore, in this contribution we consider efficient measurements only.

Let us, for a moment, focus on the average dynamics generated by this collision model. Discarding the outcome of the measurement, the instrument describes a quantum channel Λ:

(1) $$\begin{array}{c} \Lambda \left[\rho_{M}\right]=\sum_{i}I_{i}\left[\rho_{M}\right]=\sum_{i}M_{i}\;\rho_{M}\;M_{i}^{†}. \end{array}$$

The single step map for the joint state of system S and memory M is then given by

(2) $$\begin{array}{c} \Delta \Xi \left[\rho_{SM}\right]=\left(1_{S}⊗\Lambda_{M}\right)\left[W\;\rho_{SM}\;W^{†}\right]. \end{array}$$

Accordingly, the map for the *n*th step $\Xi^{\left(n\right)}$ is obtained by concatenating the single step map *n*-times:

(3) $$\Xi^{\left(n\right)}=\underset{n-\mathrm{times}}{\underset{︸}{\Delta \Xi \circ \ldots \circ \Delta \Xi }}.$$

Assuming an initial state of product form, i.e., $\rho_{SM}^{\left(0\right)}=\rho_{S}^{\left(0\right)}⊗\rho_{M}^{\left(0\right)}$, and fixing the initial state of the memory $\rho_{M}^{\left(0\right)}=\rho_{M}^{\mathrm{init}}$, we can obtain a family of completely positive and trace-preserving (CPT) maps that describe the dynamics in the system S alone by tracing over the memory after *n* steps:

(4) $$\begin{array}{c} \Gamma_{S}^{\left(n\right)}\left[\rho_{S}^{\left(0\right)}\right]={\mathrm{tr}}_{M}\left[\Xi^{\left(n\right)}\left[\rho_{S}^{\left(0\right)}⊗\rho_{M}^{\mathrm{init}}\right]\right]. \end{array}$$

It should be noted here that the reduced dynamics does not, of course, depend on how the channel Λ is implemented, nor that it is given by a measurement. In composite collision models, one commonly describes such a channel Λ by a coupling of the memory M to environmental ancillas which are traced out after their collision [1].

### 2.1. Divisibility

It is well known that the dynamics in S, as given by the dynamical map $\Gamma_{S}^{\left(n\right)}$, is in general not divisible. A family of maps $\left\{\Gamma^{\left(n\right)},n\in Z_{0}^{+}\right\}$ is said to be *completely positive (CP) divisible* if it can be decomposed as

(5) $$\begin{array}{c} \Gamma^{\left(n\right)}=\Gamma^{\left(n,m\right)}\circ \Gamma^{\left(m\right)},\;\forall m<n, \end{array}$$

where $\Gamma^{\left(n,m\right)}$ is a CPT map describing the dynamics from step *m* to step *n*. If the maps $\Gamma^{\left(n,m\right)}$ are positive but not completely positive, the dynamics is called P-divisible. If there is a $\Gamma^{\left(n,m\right)}$ which is not positive, the dynamics is called indivisible [33]. The discrete dynamical map $\Xi_{SM}^{\left(n\right)}$ for the joint state is CP-divisible by construction, since the propagator from one step to the next is given by the CPT map $\Delta \Xi_{SM}$. However, the map on the system alone $\Gamma_{S}^{\left(n\right)}$ is generally indivisible.

In this work, we will make use of a criterion to detect indivisible dynamics which has been proposed in ref. [34]. Under P-divisible dynamics, the state space volume accessible by the dynamics decreases monotonically. Thus, if this volume increases during the dynamics, the latter has to be indivisible. We briefly review this criterion in the following. The density operator ρ of a quantum system of dimension *d* can be decomposed as

(6) $$\begin{array}{c} \rho =\sum_{i=0}^{d^{2}-1}\mathrm{Tr}[\rho \;G_{i}]\;G_{i}=\sum_{i=0}^{d^{2}-1}{\vec{r}}_{i}\;G_{i}, \end{array}$$

where the $G_{1,\ldots ,d^{2}-1}$ are the Hermitian and traceless generators of the group SU(d) and $G_{0}=1/\sqrt{d}$. The vector $\vec{r}$ is called the generalised Bloch vector. The dynamical map $\Gamma_{S}^{\left(n\right)}$ can be given in the basis $\left\{G_{i}\right\}$, acting on $\vec{r}$ as

(7) $$\begin{array}{ccc} \vec{r}\left(n\right)=F^{\left(n\right)}\;\vec{r}\left(0\right), & \; & F_{ij}^{\left(n\right)}=\mathrm{Tr}[G_{i}\;\Gamma_{S}^{\left(n\right)}\left[G_{j}\right]]. \end{array}$$

Under P-divisible dynamics, the absolute value of the determinant of this matrix has to decrease during the dynamics, i.e., [35]

(8) $$\begin{array}{c} |\det F^{\left(n\right)}|\leq |\det F^{\left(m\right)}|,\;\forall n>m. \end{array}$$

Thus, an increase in $|\det F^{\left(n\right)}|$ indicates an indivisible dynamics. Such an increase has an intuitive geometrical interpretation, as it describes an inflation of the state space volume accessible by the map [34]. For two subsequent maps, we define $D_{n}=|\det F^{\left(n+1\right)}|-|\det F^{\left(n\right)}|$. We can then define a quantifier for indivisibility by summing up all positive $D_{n}$:

(9) $$\begin{array}{c} N=\sum_{D_{n}>0}D_{n}. \end{array}$$

This measure was originally proposed for a time-continuous map F(t) and then reads [34]:

(10) $$\begin{array}{c} N=\int_{\frac{\partial }{\partial t}\left|\det F\left(t\right)\right|>0}\;\frac{\partial }{\partial t}\left|\det F\left(t\right)\right|\;dt. \end{array}$$

### 2.2. Measurements and Conditional Dynamics

The measurement on the memory provides some information about the quantum state in M. Additionally, since S and M have previously interacted, the measurement can tell us something about the state of the system as well. The post-measurement joint state after the first step and an outcome $i_{1}$ for an efficient measurement reads

(11) $$\begin{array}{c} \rho_{SM}^{i_{1}}=\frac{\left(1⊗M_{i}\right)W\rho_{SM}^{\left(0\right)}W^{†}\left(1⊗M_{i}^{†}\right)}{\mathrm{tr}[\left(1⊗M_{i}^{†}M_{i}\right)W\rho_{SM}^{\left(0\right)}W^{†}]}, \end{array}$$

where $\rho_{SM}^{\left(0\right)}$ is the initial joint state. This conditional state $\rho_{SM}^{i_{1}}$ then undergoes the next step with outcome $i_{2}$ and the corresponding conditional state $\rho_{SM}^{\left(i_{1},i_{2}\right)}$, where the tuple in the superscript indicates that the state is conditioned on both outcomes $i_{1}$ and $i_{2}$. Keeping track of the outcomes also in the following steps of the collision model, we get a sequence of quantum states which is often referred to as a quantum trajectory [20,22,36,37,38,39].

For *N* steps, a trajectory is identified by an *N*-tuple of outcomes $k=(i_{1},i_{2},\ldots ,i_{N})$ with corresponding states $\left(\rho_{SM}^{i_{1}},\rho_{SM}^{\left(i_{1},i_{2}\right)},\ldots ,\rho_{SM}^{k}\right)$. In general, there are $n^{N}$ different final states $\rho_{SM}^{k}$, where *n* is the number of outcomes of a single measurement. Each of these states has a probability $p_{k}$, which is the probability of the corresponding trajectory. All these final states form the ensemble

(12) $$\begin{array}{c} E_{SM}^{N}=\left\{\rho_{SM}^{k},p_{k}\right\}. \end{array}$$

By construction, averaging over the ensemble we get the reduced joint state after *N* steps in the collision model.

(13) $$\begin{array}{c} \sum_{k}p_{k}\rho_{SM}^{k}=\rho_{SM}^{\left(N\right)}=\Xi^{\left(N\right)}\left[\rho_{SM}^{\left(0\right)}\right]. \end{array}$$

The reduced state $\rho_{SM}^{\left(N\right)}$ only depends on the initial state, the interaction *W*, and the channel Λ. However, its decomposition into an ensemble depends on the measurement operators that implement the channel Λ.

### 2.3. The Reduced Ensemble and Its Purity

Given an ensemble $E_{SM}^{N}$, we can ask what do we know about the system S alone. The reduced ensemble in S is obtained by tracing over the memory for each ensemble state:

(14) $$\begin{array}{c} E_{S}^{N}=\left\{{\mathrm{tr}}_{M}\left[\rho_{SM}^{k}\right],p_{k}\right\}=\left\{\rho_{S}^{k},p_{k}\right\}. \end{array}$$

The reduced ensemble contains everything we can know about the decomposition of the average state of the system S when we probe it by measurements on the memory system M.

It is worth noting that in a standard Markovian collision model without memory, there is always a measurement model which allows us to obtain a pure state trajectory in the system. In a composite collision model, this is generally not the case because even if the conditional states $\rho_{SM}^{k}$ are pure, they may be entangled and, thus, the reduced state in S is mixed. Therefore, we will use the average purity of the states in the reduced ensemble $E_{S}^{N}$ as a quantifier for the information one can acquire about S by measurements on M.

(15) $$\begin{array}{c} P=\sum_{k}p_{k}\mathrm{pur}\left[\rho_{S}^{k}\right]=\sum_{k}p_{k}\mathrm{tr}[{\left(\rho_{S}^{k}\right)}^{2}]. \end{array}$$

## 3. A Specific Case

Let us now consider a specific setup where both system S and memory M are qubits. We leave the interaction unitary *W* arbitrary for the moment, but fix the measurement performed on the memory M in each step to be a symmetric and informationally complete (SIC) measurement [40]. The POVM of such a measurement is given by four rank-one operators that are proportional to projectors which form a regular tetrahedron on the Bloch sphere. A possible choice is given by

(16) $$\begin{array}{ccccccc} E_{1}=\frac{1}{4}\left(1+\frac{1}{\sqrt{3}}\left(\sigma_{x}+\sigma_{y}+\sigma_{z}\right)\right), & \; & E_{2}=\sigma_{x}E_{1}\sigma_{x}, & \; & E_{3}=\sigma_{y}E_{1}\sigma_{y}, & \; & E_{4}=\sigma_{z}E_{1}\sigma_{z}. \end{array}$$

An efficient measurement implementing this POVM has the form

(17) $$\begin{array}{c} I_{i}\left[\rho_{M}\right]=\sqrt{E_{i}}\;\rho_{M}\;\sqrt{E_{i}}. \end{array}$$

One can verify that such an instrument leads on average to a depolarising channel on the memory M

(18) $$\begin{array}{c} \Lambda \left[\rho_{M}\right]=\sum_{i}I_{i}\left[\rho \right]=\sum_{i}\sqrt{E_{i}}\;\rho_{M}\;\sqrt{E_{i}}=\left(1-\lambda \right)\rho_{M}+\lambda \frac{1}{2}, \end{array}$$

with λ=2/3 being the depolarising strength. Thus, in each step, after the unitary interaction with the system S, the memory M is partially depolarised. On average, the purity of the state decreases due to the depolarisation. However, if one keeps track of the measurement outcome, the opposite is the case. After the measurement, the memory is always in a pure state corresponding to the rank-one element $E_{i}$.

### 3.1. Measurement Strength

The strong SIC measurement always results in a post-measurement joint state which is of product form. Thus, on average the measurement acts as an entanglement-breaking channel [41,42]. Let us introduce a weakened form of this measurement which does not completely cut the correlations. To this end we add a new trivial measurement operator $A_{0}$ to the instrument. With a measurement strength parameter 0≤g≤1. We then define

(19) $$\begin{array}{ccc} A_{i}=\sqrt{g\;E_{i}}, & \; & A_{0}=\sqrt{1-g}\;1. \end{array}$$

One can see this measurement as a probabilistic implementation of the strong measurement with probability *g*. With probability (1−g) no measurement is performed and the trivial operator $A_{0}$ is applied. The measurement still implements a depolarising channel but the degree of depolarisation now depends on *g*:

(20) $$\begin{array}{c} \Lambda_{g}\left[\rho \right]=\left(1-\frac{2g}{3}\right)\rho +\left(\frac{2g}{3}\right)\frac{1}{2}=\left(1-\lambda_{A}\right)\rho +\lambda_{A}\frac{1}{2}, \end{array}$$

which shows that the measurement strength *g* tunes between the strong case ($g=1,\lambda_{A}=2\;/\;3$) given in Equation (18) and the trivial case ($g=0,\lambda_{A}=0$) where no measurement is implemented and the channel is just the identity.

### 3.2. Divisibility

Before we investigate the ensemble purities and the information gain for different measurement strengths *g* we briefly comment on the divisibility of the discrete dynamical map $\Gamma_{S}^{\left(n\right)}$ on the system. In general the divisibility will depend on the unitary interaction gate *W*. Here, we are interested in the generic behaviour of this model. Therefore, we randomly sample collision models which only differ in their interaction gate *W*. This unitary gate is randomly drawn from the Haar measure [43]. Note that the gate *W* is only sampled once, but is then kept fixed during the steps of the collision model. We propagate the model until the reduced state in the system S reaches a steady state and calculate the indivisibility N in Equation (9) to check the P-divisibility of the dynamics.

**Figure 2:**
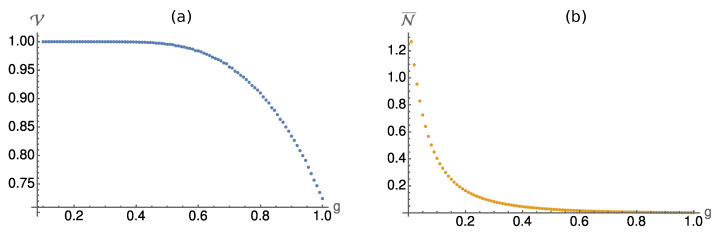
(**a**) The plot shows the fraction of unitaries *W* which lead to indivisible dynamics in dependence of the measurement strength *g*. For weak measurements, almost all *W* lead to indivisibility. For larger *g*, the ratio decreases. However, it has to be stressed that even in the limit g→1, where the channel Λ is entanglement breaking in each step, more than half of the unitaries *W* lead to indivisible dynamics. (**b**) The average of the indivisibility quantifier N (see Equation (9)) is plotted. The plot shows that the average values of N quickly decrease with increasing measurement strength *g*. Thus, even though many *W* lead to indivisible dynamics also for large *g*, those will hardly be distinguishable from a P-divisible dynamics with the measure N.

By sampling sufficiently many unitaries *W* from the Haar measure, we can estimate the ratio V between the volume of unitaries which lead to indivisible dynamics and the volume of all possible unitaries. The results are plotted in Figure 2. As one might expect, for small measurement strengths, i.e., g→0, almost any *W* leads to indivisible dynamics. Interestingly, in the strong measurement limit, i.e., g→1, where the measurement is entanglement breaking, still more than half of the gates *W* generate indivisible dynamics. This demonstrates yet again that entanglement between the system and an environment is not necessary for a non-Markovian behaviour. The joint state of S and M is separable at all steps (cf. [44]).

Furthermore, we plot the average $\bar{N}$ of the indivisibility measure N. This curve shows that, even though most of the dynamics are indivisible, for larger *g* the dynamics are very close to dynamics for which P-divisibility cannot be detected.

### 3.3. Different Measurements

The reduced dynamics in the system S and its P-divisibility does not depend on the specific implementation of the depolarising channel. However, if one does not discard the measurement outcomes but uses them to determine conditional dynamics, the specific implementation of $\Lambda_{g}$ plays a role and the instrument defined in Equation (19) is not the only choice. In fact, there are infinitely many possible decompositions. In this article, we consider two further measurements which are qualitatively different from measurement *A*. We then investigate their different behaviours with respect to the information one can obtain about the conditional states in the system, measured in terms of the ensemble purity P.

To construct the weakened measurement *A*, we have added an element $A_{0}$ proportional to the identity, which tells us that nothing happened to the memory. Instead of adding the identity as an additional measurement element, we can construct unsharp versions of the original strong measurement operators $E_{i}$:

(21) $$\begin{array}{c} B_{i}=\sqrt{fE_{i}+\left(1-f\right)\frac{1}{4}}. \end{array}$$

Here, none of the measurement operators are of rank one, implying that this measurement never leads to pure product states. As a third measurement we introduce the operators

(22) $$\begin{array}{ccc} C_{i}=U_{i}\sqrt{h\;E_{i}}, & \; & C_{0}=\sqrt{1-h}\;1, \end{array}$$

where $U_{i}$ is the unitary that flips the rank-1 element $E_{i}$ to its orthogonal which lies on the opposite side of the Bloch sphere. This instrument is structurally similar to measurement *A*, and could be implemented by first performing measurement *A* and subsequently applying a unitary gate depending on the outcome *i*.

Both measurements *B* and *C* lead to a depolarizing channel with the depolarisation strengths $\lambda_{B}=2\;/\;3(1-\sqrt{1-f^{2}})$ and $\lambda_{C}=3h\;/\;4$, respectively. We require that all measurements generate the same channel $\Lambda_{g}$, so we have to set

(23) $$\begin{array}{ccc} f=\sqrt{2g-g^{2}}, & \; & h=\frac{g}{2}. \end{array}$$

The reduced dynamics of system S and memory M is independent of the chosen measurement. However, the information one can obtain about the conditional states in S substantially depends on the implemented measurement.

### 3.4. The Corresponding POVMs

By construction, the different measurements lead to the same channel $\Lambda_{g}$. However, it has to be stressed that the measured POVMs differ. The POVM implemented by measurement *A* consists of five elements:

(24) $$\begin{array}{ccc} A_{i}^{†}A_{i}=g\;E_{i}, & \; & A_{0}^{†}A_{0}=\left(1-g\right)\;1. \end{array}$$

The third measurement *C* generates a similar POVM:

(25) $$\begin{array}{ccc} C_{i}^{†}C_{i}=h\;E_{i}=\frac{g}{2}\;E_{i}, & \; & C_{0}^{†}C_{0}=\left(1-h\right)\;1=\left(1-\frac{g}{2}\right)\;1. \end{array}$$

However, since h=g/2, the POVM corresponding to measurement *C* is less informative than the one corresponding to measurement *A*. We will see below that this has an important impact on the conditional dynamics.

In contrast to the cases *A* and *C*, the POVM corresponding to the second measurement *B* has only four different elements

(26) $$\begin{array}{c} B_{i}^{†}B_{i}=fE_{i}+\left(1-f\right)\frac{1}{4}=\sqrt{2g-g^{2}}E_{i}+\left(1-\sqrt{2g-g^{2}}\right)\frac{1}{4} \end{array}$$

For 0<g<1, we have $f=\sqrt{2g-g^{2}}>g$, and thus, the strong POVM elements $E_{i}$ appear with a higher weight in measurement *B* than in measurement *A*. Therefore, one can expect that measurement *B* is the most informative one.

**Figure 3:**
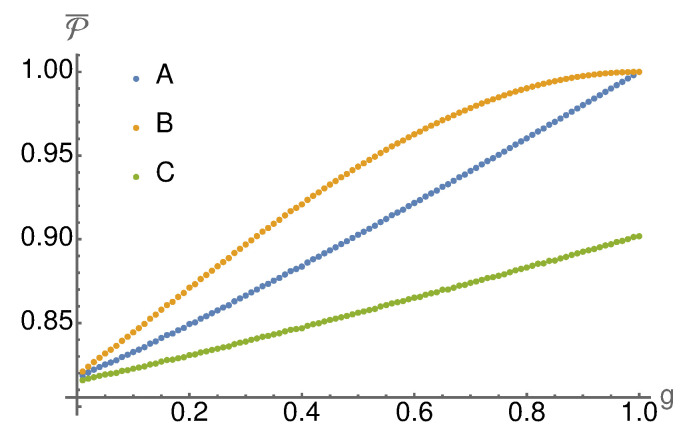
We plot the average purity of the steady state ensembles averaged over random unitary interaction gates *W* (in each run the gate is fixed for all steps in the collision model). As expected, one can see that the purity increases with the measurement strength. Furthermore, the better performance of measurement *B* is a generic feature.

To show this, we analyse the conditional ensembles $E_{S}^{N}$ in the steady-state limit, i.e., N→∞. We numerically calculate the ensemble purity P from Equation (15) over unitaries *W* from the Haar measure and plot in Figure 3 the average purity $\bar{P}$ as a function of the measurement strength *g*. As expected, measurement *B* leads to higher ensemble purities than measurement *A* and measurement *C* performs worse. In the weak measurement limit (g→0), all three agree. Measurement *A* and *B* also agree in the strong measurement limit (g→1), which is not surprising because both converge to the same instrument in this limit (c.f. Equations (19) and (21)).

It is worth noting that the trajectories of the joint state of S and M are pure for any of the measurements and for any g>0. Thus, the lack of purity in the reduced ensemble in S stems from the fact that the joint states are entangled on average.

## 4. Time-Continuous Limit

So far, we only considered a discrete model. We now study the continuous limit and demonstrate that the behaviour observed in the discrete case similarly holds. We can approximate such dynamics by looking at short time intervals Δt. The interaction gate *W* is generated by a Hamiltonian *H*. During a time interval Δt, the transformation reads to first order in Δt

(27) $$\begin{array}{c} W=1-i\Delta t\;H. \end{array}$$

In order to obtain a time-continuous limit, the subsequent measurement has to result in a channel whose effect on the state is also of order Δt. In other words, the measurement strength needs to scale with the time step as g∝Δt. Looking at the form of the resulting channel in Equation (20) we set, for convenience, g=3/2γΔt, where gamma is a positive real constant describing the strength of the measurement. In a single time step, the joint state $\rho_{SM}$ changes to first order in Δt as

(28) $$\begin{array}{cc} \rho_{SM}\left(t+\Delta t\right) & =\left(1⊗\Lambda_{g}\right)\left[W\;\rho_{SM}\left(t\right)\;W^{†}\right]\\ \; & =\rho_{SM}\left(t\right)-i\left[H,\rho_{SM}\left(t\right)\right]\Delta t-\gamma \Delta t\left(\rho_{SM}\left(t\right)-{\mathrm{tr}}_{M}\left[\rho_{SM}\right]⊗\frac{1_{M}}{2}\right). \end{array}$$

Thus, in a time-continuous limit the joint state evolves according to the following differential equation.

(29) $$\begin{array}{cc} \; & \lim_{\Delta t\to 0}\frac{\rho_{SM}\left(t+\Delta t\right)-\rho_{SM}\left(t\right)}{\Delta t}={\dot{\rho }}_{SM}\left(t\right)\\ \; & =-i\left[H,\rho_{SM}\left(t\right)\right]-\gamma \left(\rho_{SM}\left(t\right)-{\mathrm{tr}}_{M}\left[\rho_{SM}\left(t\right)\right]⊗\frac{1_{M}}{2}\right). \end{array}$$

It contains a unitary part governed by the interaction Hamiltonian *H* and the depolarising process on M.

In order to investigate the generic behaviour of this continuous model, we sample the unitary gate *W* through its Hamiltonian *H*. These are drawn from the Gaussian unitary ensemble (GUE) [45]. To this end, we generate two real matrices *S* and *T* with all entries being randomly chosen from a normal distribution with mean μ=0 and width σ=1. The matrix $H=[{\left(S+iT\right)}^{†}+\left(S+iT\right)]/2$ is a Hamiltonian belonging to the GUE [46].

### 4.1. Divisibility

**Figure 4:**
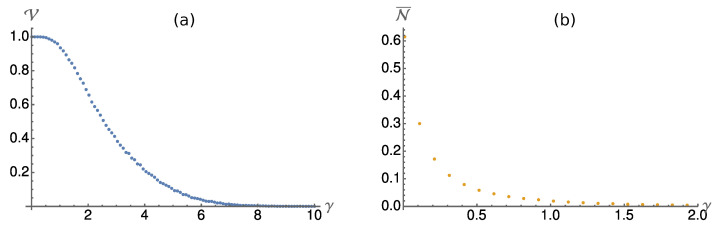
(**a**) We plot the fraction V of Hamiltonians in the Gaussian unitary ensemble (σ=1) that lead to indivisible dynamics. For small measurement strength (weak depolarisation), almost all Hamiltonians lead to indivisible dynamics. For stronger measurements, more and more Hamiltonians generate divisible dynamics. (**b**) The average divisibility (see Equation (10)) shows a steep decrease with increasing measurement strength γ. Already at γ=2, where still more than 50% of the Hamiltonians lead to indivisible dynamics, the average divisibility has dropped to a value below $\bar{N}=0.01$, so most of the dynamics are hard to distinguish from P-divisible ones.

In the time-continuous model, the divisibility increases with increasing measurement strength γ, too. In Figure 4a, we plot the fraction V of the GUE for which the collision model leads to indivisible dynamics. Again, for small measurement strengths, almost all Hamiltonians lead to indivisible dynamics. For large γ, the dynamics are almost always divisible, as can be expected, since in this regime the strong depolarisation suppresses possible revivals in the system dynamics. The average over the indivisibility N, as defined in Equation (10), is plotted in Figure 4b. As in the discrete case, $\bar{N}$ quickly drops with increasing measurement strength γ.

### 4.2. Ensemble Purity

As in the discrete case, we can implement the depolarisation on M using the three different instruments A,B,C. The three measurement schemes are obtained by plugging g=3γΔt/2 into the Equations (19), (21) and (22).

**Figure 5:**
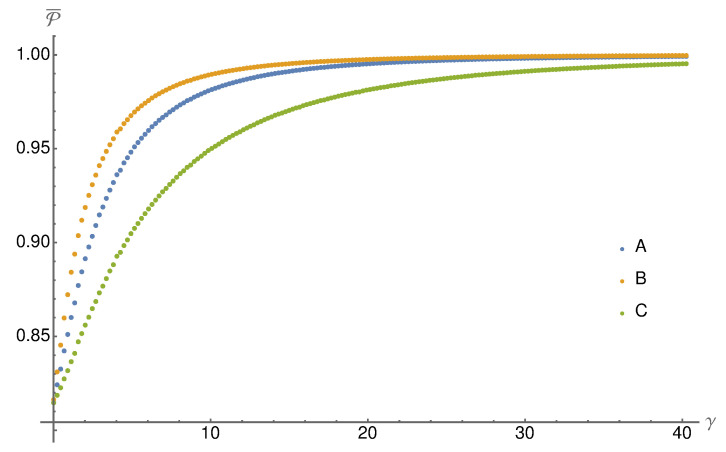
The average ensemble purity $\bar{P}$ for the three different measurements (see Equations (19), (21) and (22)) is plotted in dependence of the measurement strength γ. The average is taken over interaction Hamiltonians from the Gaussian unitary ensemble (σ=1). As in the discrete model, measurement *B* leads to the most pure ensembles, followed by *A* and *C*. All three measurement scenarios lead to pure ensembles in the strong measurement limit γ→∞.

In Figure 5 we plot the ensemble purity averaged over the random Hamiltonians sampled from the GUE. As in the discrete case, measurement *B* performs better than *A* and *C*. In the weak measurement limit γ→0, all measurements perform equally. In contrast to the discrete case, now measurement *C* can also produce pure ensembles in the strong measurement limit γ→∞. However, the necessary strength is much larger than for the other two measurements. Thus, on average, measurement *B* indeed leads to the most pure trajectories and ensembles in the system S.

## 5. Conclusions

In this article, we have investigated quantum trajectories in composite collision models. While standard Markov collision models always allow for a measurement model that produces pure state trajectories, this is generally not the case in collision models with memory due to possible entanglement between the system and the memory, even in the measured quantum trajectories. We have analysed a two-qubit model where the memory undergoes a depolarising channel on average. This channel can be implemented as a measurement of a SIC observable. By introducing a weakened form of this measurement, we have shown that the divisibility of the resulting dynamics of the system S depends on the measurement strength.

We have investigated three different measurements which all lead to the same dynamical map but to different quantum trajectories. While the joint trajectories of system S and M are in general pure, the reduced ensembles in S alone are not. The purity of the latter strongly depends on the measurement performed on the memory M. To draw conclusions about a generic case, we determine the divisibilities and the ensemble purities for randomly sampled unitary interaction gates *W* in the composite collision models and estimate the quantities as averages over the Haar measure. The model can be extended to a time-continuous case where the measurement strength becomes infinitesimal. To investigate the generic behaviour, the averaging is now taken over interaction Hamiltonians sampled from the Gaussian unitary ensemble. The qualitative differences between the measurements also persist in the continuous case.

For Markov evolutions, it is well understood how different measurements lead to different unravellings of the dynamics. In particular, pure state unravellings always exist. For dynamics with memory effects, many questions related to quantum trajectories remain open. In our contribution, we show how composite collision models can be used to gain an understanding of why physically valid quantum trajectories are not necessarily pure. Our findings show that different measurements on the memory part can reveal a different amount of information about the state of the system. The different performances do not depend on a fine tuning of the measurement with respect to the interaction, but are generic.

A qualitative relationship between the divisibility and the achievable ensemble purity becomes apparent. On the one hand, stronger measurements allow for a higher purity in the trajectories, but on the other hand they also lead to a faster depolarisation of the memory M and therefore increase the divisibility. Thus, there is a trade-off between the non-Markovian behaviour of the system dynamics and the knowledge one can gain about the systems state. However, it has to be stressed that even though this interplay is a generic feature, as can be seen from our simulations, a more quantitative relation is still missing.

For a better understanding of physical quantum trajectories in a non-Markovian system, optimality conditions for the performed measurements would be desirable. Such an optimal choice will always depend on the figure of merit one is interested in. Composite collision models can provide a powerful framework for these questions because the memory effects are transparently included into the setup. The model is not restricted to the depolarising case investigated here. Furthermore, the homogeneous form of the measurement scenario is a strong restriction. Adaptive measurements, i.e., measurements, where the instrument in the next step depends on the previous outcomes, could further improve the scheme.
